# Supplementary material for: The Relation of the Iron Metabolism Index to the Vulnerability Index of Carotid Plaque with Different Degrees of Stenosis
Source: Diagnostics (Basel). 2023 Oct 12;13(20):3196. doi: 10.3390/diagnostics13203196 (PMC10606419; doi:10.3390/diagnostics13203196)
Supplement: Supplementary file 1 [file diagnostics-13-03196-s001.zip › diagnostics-2557711-supplementary.pdf]

Supplementary Materials

The Relation of the Iron Metabolism Index to the Vulnerability Index of Carotid Plaque with Different Degrees of Stenosis

Methods for measuring indicators of serum iron metabolism

Within 2 hours after blood collection, the blood samples were centrifuged at 3000 Rpm for 20 minutes at room temperature to obtain serum and plasma for further detection. Kits for the detection of indicators of iron metabolism, Biochemistry Analyzer (cobase c702) and Electrochemiluminescence Immunoassay Analyzer (cobase e601) are all from Roche Diagnostics GmbH.

Serum ferritin (SF) concentration was detected by electrochemiluminescence immunoassay. Serum transferrin receptor (sTfR) was measured by immunoturbidimetric assay. Serum iron concentration and unsaturated iron binding capacity (UIBC) were detected by colorimetric assay. Total iron binding capacity (TIBC) is the sum of serum iron concentration and unsaturated iron binding capacity.

Immunohistochemical staining procedures

Carotid plaques were collected immediately after CEA, fixed in 4% paraformaldehyde and embedded in paraffin. The slices were cut at 2 μm intervals. After dewaxing using xylene and dehydration using gradient alcohol, antigen repair with EDTA and endogenous peroxidase inactivation using hydrogen peroxide were performed. Sections were blocked with serum and then the sections were exposed to the following primary antibodies: monoclonal rabbit anti-ferritin heavy chain (clone EPR3005Y; 1:300; Abcam; UK), monoclonal mouse anti-ferritin light polypeptide (clone 1F9F5; 1:200; Proteintech; US), monoclonal rabbit anti-transferrin receptor (clone EPR20584; 1:500; Abcam; UK) and monoclonal mouse anti-human CD68 (clone CD68, PG-M1; 1:100; GeneTech; Shanghai, China). The sections were incubated with secondary antibodies, and then color was developed with DAB solution. The sections were restained with hematoxylin solution, dehydrated with a gradient of ethanol, cleared with xylene and sealed with neutral resin.

Table S1. Summary of Multiple Linear Regression Model results.

R	R^2	Adjusted R^2	P-value (ANOVA Significance.)
0.630	0.397	0.326	0.014

ANOVA = Analysis of Variance.
